# Phenotypic Expression of Autoimmunity in Children With Autoimmune Thyroid Disorders

**DOI:** 10.3389/fendo.2019.00476

**Published:** 2019-07-12

**Authors:** Tommaso Aversa, Domenico Corica, Giuseppina Zirilli, Giovanni Battista Pajno, Giuseppina Salzano, Filippo De Luca, Malgorzata Wasniewska

**Affiliations:** Department of Human Pathology of Adulthood and Childhood, Unit of Pediatrics, University of Messina, Messina, Italy

**Keywords:** additional autoimmunity, extra-thyroidal autoimmunity, Graves' disease, Hashimoto's thyroiditis, metamorphic autoimmunity

## Abstract

Autoimmune thyroid diseases (AITDs), including Hashimoto's thyroiditis (HT) and Graves' disease (GD), tend to aggregate with other non-thyroidal autoimmune diseases (NTADs). Aim of this Mini-review is to report the most recent insights concerning the clustering of NTADs in pediatric patients with either HT or GD, the pathophysiology of AITDs and the metamorphic thyroid autoimmunity. A systematic literature research of the last 15 years, according to EQUATOR statement, was carried out through MEDLINE via PubMed (http://www.ncbi.nlm.nih.gov/pubmed/) Embase, CINAHL, Cochrane Library, based on the following keywords: (autoimmune thyroid disease OR Hashimoto thyroiditis OR Grave's disease) AND (autoimmune comorbidities OR extra-thyroidal autoimmune disorders) AND (children OR adolescents OR pediatrics) AND (celiac disease OR type 1 diabetes mellitus OR arthropathies OR cutaneous diseases) AND (Turner syndrome OR Down syndrome). One-hundred and twenty-eight manuscripts were extrapolated but only seventeen were eligible. On the basis of the available reports it may be inferred that clustering of NTADs can be significantly modified by both patients' age at AITDs presentation and association with Down's syndrome (DS). Particularly, the association of AITDs with celiac disease and type 1 diabetes was most commonly reported in children than in adults. A sequential shifting from HT to GD has been described in children with AITDs, and it seems to be more frequent in children with DS than in those without DS. Coexistence of autoimmune diseases might be the result of a complex interaction among genetics, environment and epigenetic modifications that are able to affect gene expression, immune system response and, finally, the pathogenesis of autoimmune diseases.

## Background

Autoimmune diseases (ADs) represent a heterogeneous group of chronic disorders, which afflict specific target organs or multiple organ systems and are initiated by the loss of immunological tolerance to self-antigens. Two peculiar aspects of these disorders are that more ADs tend to aggregate in the same patient (polyautoimmunity) and that affected individuals tend to cluster in the same nuclear family (familial autoimmunity). Such shared characteristics suggest that the development of ADs is affected by similar genetic, epigenetic and environmental factors (1).

Autoimmune thyroid diseases (AITDs), which includes both Hashimoto's thyroiditis (HT) and Graves' disease (GD), affects an estimated 5% of the general population, making it one of the most prevalent ADs (2). The concept of autoimmune diathesis is widely accepted also for AITDs, since previous studies documented that individuals with AITDs have an increased relative risk of developing a picture of polyautoimmunity, thus highlighting the role of a genetic shared predisposition for many ADs (3–5). Recent reports have evidenced that the clustering of different non-thyroidal autoimmune diseases (NTADs) in patients with AITDs may be conditioned also by other intrinsic factors, such as age (6) and association with specific chromosomopathies (7–9), apart from genetic predisposition.

Other peculiarities of thyroid autoimmunity are represented by the links between HT and GD and the fluctuations in thyroid function from hypo- to hyperthyroidism or viceversa, that may sometimes occur in patients with AITDs (10, 11).

Aim of this Mini-review is to report the most recent insights on the metamorphic thyroid autoimmunity and the aggregation between either HT or GD and NTADs in patients with AITDs.

## Methods

A systematic research, according to EQUATOR statement (12, 13), was carried out through MEDLINE via PubMed (http://www.ncbi.nlm.nih.gov/pubmed/) Embase, CINAHL, Cochrane Library, from January 2004 to March 2019, based on the following keywords: (autoimmune thyroid disease OR Hashimoto thyroiditis OR Grave's disease) AND (autoimmune comorbidities OR extra-thyroidal autoimmune disorders) AND (children OR adolescents OR pediatrics) AND (coeliac disease OR type 1 diabetes mellitus OR arthropathies OR cutaneous diseases) AND (Turner syndrome OR Down syndrome).

## Results

One-hundred and twenty-eight manuscripts were screened and evaluated. Finally, seventeen manuscripts satisfying the following eligibility criteria have been selected: articles written in English belonging to the categories of clinical trial, observational study, Meta-Analysis, multicenter Study, randomized controlled trial, review, concerning the association between AITDs and NTADs in children and adolescents with HT or GD (Tables 1, 2), the pathophysiology of AITDs and the metamorphic thyroid autoimmunity.

**Table 1:** Studies evaluating the association between autoimmune thyroid diseases (AITDs) and non-thyroidal autoimmune diseases (NTADs) in children and adolescents with Hashimoto's thyroiditis and Graves' disease.

	N^**°**^ of patients, type of AITDs, and age at evaluation^*^	**Frequency of coexistence of AITDs and NTADs (%)**	**Frequency of associated NTADs (%)**
**HASHIMOTO'S THYROIDITIS AND NTADs**			
Ruggeri et al. (6)	553 HT (11.11 ± 2.96 years)	18.8	7.2% CD 6.9% T1DM 2.7% Vitiligo 0.7% AD 0.18% RA 0.18% Vasculitis
Sattar et al. (14)	302 HT–GD (3.1–24.9 years)	6.6	4.3% T1DM with HT 1.9% CD with HT
Gómez López et al. (15)	29 HT (11; 5–15 years)	13.7	6.9% CD 3.4% Alopecia 3.4% Vitiligo
Aversa et al. (8)	146 HT with DS (6.5 ± 4.8 years)	58.2	11.4% Alopecia 11% CD 3.4% T1DM
	553 HT controls (11.1 ± 3.0 years)	18.8	7.2% CD 6.9% T1DM 0.9% Alopecia
Aversa et al. (9)	174 HT with DS (6.1; 1–18 years)	56.3	27% Alopecia 14.3% CD 13.2% Vitiligo 4% T1DM
	678 HT controls (11.2; 2.5–18 years)	17.8	6.9% T1DM 6% CD 2.8% Vitiligo 0.9% Alopecia
Aversa et al. (16)	12 HT–GD with DS (3–13.5 years)	12	41.6% CD 25% T1DM 8.3% RA
**GRAVES' DISEASE AND NTADs**			
Valenzise et al. (7)	7 GD with TS (14.6; 9.8–29.5)	57.1	14.3% T1DM 14.3% CD 4.5% T1DM
	89 GD controls (11.3; 3.4–17.9)	11.2	4.5% Vitiligo 1.1% RA 1.1% Alopecia
De Luca et al. (17)	28 GD with DS (9.9 ± 4.4 years)	32.1	32.1% CD 7.1% T1DM 3.5% RA 3.5% AD 4.6% Vitiligo
	109 GD controls (11.5 ± 3.5 years)	12.8	3.7% T1DM 2.7% CD 0.9% Alopecia 0.9% AG 0.9% RA
Sattar et al. (14)	302 HT–GD (3.1–24.9 years)	6.6	0.33% CD with GD and DS

* *Age of patients are reported in mean age ± Standard deviation or median and range, as in the articles*.

*AITDs, Autoimmune thyroid diseases; NTADs, non-thyroidal autoimmune diseases; HT, Hashimoto thyroiditis; GD, Graves' disease; T1DM, Type 1 diabetes mellitus; RA, Rheumatoid arthritis; AD, Addison disease; CD, celiac disease; AG, atrophic gastritis; DS, Down's syndrome; TS, Turner syndrome*.

**Table 2:** Studies evaluating the association between non-thyroideal autoimmune diseases (NTADs) and autoimmune thyroid diseases (AITDs) in children and adolescents with celiac disease and type 1 diabetes mellitus.

	N^**°**^ of patients, type of AITDs, and age at evaluation^*^	**Primary evaluated AD**	**Coexistence of ADs (%)**	**Associated AITDs (%)**
**NATDs AND AITDs**				
Størdal et al. (18)	3,006 CD (0–12 years)	CD	6.1	1.4% AITDs
Canova et al. (19)	1,215 CD (6; 0–23 years)	CD	8.4	4.8% AITDs
Diamanti et al. (20)	558 CD (10.3; 0.8–11.2)	CD	12	10% AITDs
van der Pals et al. (21)	335 CD (12-years old children)	CD	7.7	7.7% AITDs
Elfstrom et al. (22)	9,338 CD (0–15 years)	CD	0.6	0.6% AITDs
Cosnes et al. (23)	378 CD (<16 years)	CD	16.9	1.8% AITDs
Lombardo et al. (24)	1,323 T1DM (5.2 ± 3.0 years)	T1DM	0.5	0.5% GD
Riquetto et al. (25)	233 T1DM (11.9 ± 3.3 years)	T1DM	21	21% AITDs
Aitken et al. (26)	136 T1DM with DS	T1DM	67.6	50% AITDs

* *Age of patients are reported in mean age ± Standard deviation or median and/or range, as in the articles*.

*AITDs, Autoimmune thyroid diseases; NTADs, non-thyroidal autoimmune diseases*;

*ADs, autoimmune diseases; T1DM, Type 1 diabetes mellitus; CD, celiac disease; GD, Graves' disease*.

## Discussion

### Clustering of Different NTADs in Patients With HT

HT is worldwide the most common AITDs at any age and its incidence seems to be increased in the last decades (6). This disorder generally proceeds from an underlying autoimmune diathesis, featured by anti-thyroid autoantibody production, toward different presentation patterns: more often subclinical or overt hypothyroidism, more rarely subclinical or overt hyperthyroidism (11, 27–29).

**Figure 1:**
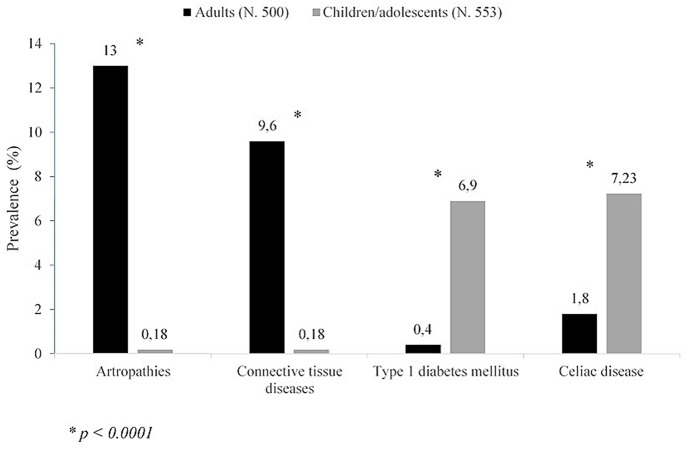
Prevalence of non-thyroidal autoimmune diseases in either adults or children/adolescents with Hashimoto's thyroiditis (according to the results of (6) of this study).

A wide spectrum of NTADs, ranging from organ-specific to systemic diseases, has been variously described in association with HT (3, 4, 6, 26, 30–34). According to the results of a very recent study, covering a population of 1,053 adults and children with HT, the NTADs that are most frequently encountered in HT patients are, in decreasing order: arthropathies (6.3%), cutaneous diseases (5.1%), connective diseases and coeliac disease CD (4.6%, respectively), type 1 diabetes mellitus T1DM (3.8%) and Addison's disease (0.9%). However, according to that report, the clustering of ADs may be significantly conditioned by patients' age at the time of HT presentation (6). In fact, in adults the most frequently associated illnesses were found to be arthropathies and connective tissue diseases (Figure 1). On the contrary, in children and adolescents these disorders were absent or very rare and the most frequent ones were T1DM and CD (Figure 1). Skin diseases were detected with similar prevalence in both adults and children, vitiligo being the commonest one (6). On the basis of these results it was inferred that different types of NTADs should be investigated in relation with patients' age (6), since some NTADs tend to occur early in life (i.e., T1DM and CD), whilst others tend to occur later (i.e., arthropathies and connective tissue diseases) (6). Other interesting results from that study were that both the prevalence of associated NTADs and the number of patients suffering from two or more NTADs were higher in adults than in children. Furthermore, the individuals with associated NTADs were significantly older than the ones without comorbidities. On the light of these data, it was concluded by those authors that adults with newly diagnosed HT may be more exposed to the risk of developing autoimmune comorbidities, if compared to young patients with early-onset HT (6). Therefore, an advanced age at HT presentation *per se* seems to be able to condition a different clustering of NTADs and a more severe expression of autoimmunity in individuals with the same AITD (namely HT) (6). A possible explanation to autoimmunity increase with aging is the reduction of immune tolerance and, generally, a decline of immune system integrity involving both innate and adaptive immune responses, a process known as *immunosenescence* (35). Notwithstanding the several pathways involved in immune tolerance are not completely clarified, interesting results have been reported in a recent study, in which Duggal et al. (36) suggested that an age-related decline in number and function of specific subtypes of B cell, and the consequent reduction of their regulatory role in immune response, may contribute to the reduced immune tolerance and increased autoimmunity seen with aging (36).

Most of the studies involving pediatric cohort reported the association of HT with CD and/or T1DM (8, 9, 14–16) (Table 1); viceversa, AITDs onset has been frequently reported in patients affected by T1DM or CD (18–26) (Table 2). Sattar et al. (14), in a large population of children and young adults (91% of patients were ≤ 17 years) affected by AITDs (HT in 95% of patients), reported a higher prevalence of positive transglutaminase-IgA titers and of biopsy-confirmed CD (2.3%) in patients with AITDs compared to the healthy US population (Table 1). In a cohort of children affected by CD, Canova et al. (19) documented a prevalence of 4.8% of AITDs. These authors demonstrated an increased risk of developing autoimmune hypothyroidism in CD subjects (HR 4.64, 95% CI 2.88–7.46) and, on the other hand, that the condition of autoimmune hypothyroidism is a strong risk factor for CD (OR 6.55, 95% CI 3.61–11.89) (Table 2).

Another intrinsic variable, which seems to be able to modify the aggregation of NTADs in patients with HT, is the possible association with Down's syndrome (DS), that is one of the most common chromosomal disorders. Children with DS are known to have an increased predisposition to thyroid, gut and islet autoimmunity (26).

Phenotypic expression of HT has been demonstrated to be significantly affected by the association with DS, in terms of both epidemiology and course (8, 37, 38). Furthermore, according to the results of another report, the link with DS might be able to modify the aggregation of extra-thyroidal ADs that is generally observed in children with HT but without DS, by favoring the clustering of alopecia areata and vitiligo, irrespective of age, and even CD, at least in the sub-cohort of patients aged ≥6 years (9). Such a peculiar expression of autoimmunity in patients with DS might be due to increased expression of AIRE gene, which could, in turn, interfere with autoimmune regulation (39).

Turner syndrome (TS) is another common chromosomopathy, that has been reported to be associated with an increased risk of various ADs, but especially HT and CD (40, 41). The link with TS has been shown to have negative repercussions on the evolution over time of thyroid function in girls with TS (37, 38, 42, 43), which suggested the opportunity of a strict monitoring of thyroid tests in these patients, in order to recognize at a proper time a possible deterioration from euthyroidism to hypo- or hyperthyroidism (42, 43). The association with TS, however, seems that it is not able to modify the clustering of NTADs in the girls with HT (42).

### Clustering of NTADs in Patients With GD

It is unclear whether HT and GD, often referred to as AITDs, aggregate to the same extent with other ADs. It has to be considered, however, that GD is much less common than HT, especially in pediatric age. Therefore, it is not surprising that the available data about the clustering of NTADs in children with GD are not very numerous (Tables 1, 2).

Some authors specifically addressed the question whether differences exist in association with other autoimmune illnesses and/or autoantibodies between HT and GD (44). According to the results of that study, 359 adult patients with HT showed a two- to three-fold greater risk for adrenal ADs and antibodies than 523 adult patients with GD (44). Furthermore, combined clustering of adrenal and β-cell autoimmunity and combined clustering of gastric and adrenal autoimmunity were both seen more often in HT patients, whereas aggregation with CD was low in both groups of patients (44). These data indicate that HT and GD differ in their clinical expression regarding additional autoimmunity, which gives evidence against the indiscriminate use of AITD as a single entity (44).

According to the results of another important study, covering 2,791 patients with GD, the most common associated AD was rheumatoid arthritis (3.2%), followed by vitiligo and pernicious anemia (1.4%, respectively), T1DM (1.1%), inflammatory bowel disease (1.0%), and CD (0.9%) (3). In the analysis of those results, it has to be considered that the mean age of GD patients in that series was 43 years, which can explain the relatively elevated prevalence of rheumatoid arthritis and pernicious anemia. In fact, it is known that some associated ADs tend to present early in life, whereas others tend to occur later (45).

Another interesting aspect, which has been just recently evidenced in a very large population of 3,209 adult patients with GD, was that the 984 individuals with Graves' ophthalmopathy exhibited another AD more frequently than those without ophthalmopathy (46). Interestingly, in this very recent study the prevalence of GD patients with another associated AD (16.7%) was distinctly higher than that previously reported in other adult populations with GD: respectively 6.5% (44) and 9.7% (3).

Due to the low prevalence of GD in childhood and adolescence, pediatric studied on the prevalence of autoimmune comorbidities in young patients with GD are few and based on limited study cohorts (Tables 1, 2). In a series of 109 children and adolescents aged between 3.4 and 17.9 years, it was found that the most common associated AD was vitiligo (4.6%), followed by T1DM (3.7%), CD (2.7%), alopecia, atrophic gastritis, and rheumatoid arthritis (0.9%, respectively) (17). The prevalence of associated NTADs detected in that population confirms that patients' age plays a key-role in conditioning the clustering of extra-thyroidal ADs in patients with AITDs (3, 6).

While T1DM is known to occur relatively often in children with GD, on the contrary GD prevalence in children with T1DM has been described to be almost identical to that reported in the general pediatric population, which suggests that screening programs based on periodical thyrotropin receptor autoantibody assessments are not useful in T1DM children and adolescents (24).

Coexistence of ADs is the result of a complex interaction among environmental and genetics factors. Concerning the genetic susceptibility, human leukocyte antigen (HLA) and Cytotoxic T-Lymphocyte Antigen 4 (CTLA-4) polymorphisms have been reported to be related with both T1DM and AITDs (47–50). In a large cohort of subjects affected by T1DM and AITDs, Einarsdottir et al. (47) suggested a strong interaction of HLA and the CTLA4 region in conferring susceptibility to ADs. In particular, Moriguchi et al. (51) demonstrated an association between HLA and glutamic acid decarboxylase autoantibodies (GAD Ab) positivity in subjects affected by AITDs, especially in those with GD. These authors suggested that this association confers susceptibility to develop β-cell autoimmunity and T1DM in AITDs patients, particularly in GD and in those ones with a high titer of GAD Ab.

Moreover, given the stronger association between CTLA4 polymorphisms with AITDs than with T1DM (49), Ikegami et al. (50), demonstrating a stronger association of CTLA4 with T1DM complicated with AITDs than with T1DM without AITDs, suggested the possibility that the linkage between CTLA4 and T1DM could be due to the association of the CTLA4 polymorphism with coexisting AITDs (50). Furthermore, these authors reported that CTLA4 itself is affected by HLA genotype, documenting a stronger association between CTLA4 and HLA in patients with very high-risk genotypes compared to those with other genotypes (50).

Also in GD children, as well as in those with HT (9), phenotypic expression of additional autoimmunity can be significantly modified by the association with DS. In fact, the prevalence of children with associated NTADs reported by De Luca et al. (17) was distinctly higher in young patients with DS (32.1%) than in those without this chromosomopathy (12.8%). Furthermore, whereas 33.3% of children with DS and associated NTADs were affected by at least two extra-thyroidal disorders, all the ones without DS exhibited only one additional AD (17). In the group with DS the most frequent NTAD was CD (28.6%), whereas in the group without DS the AD which was most commonly associated with GD was vitiligo (17).

### Pathophysiology of AITDs and Metamorphic Thyroid Autoimmunity

Both HT and GD arise from a complex interplay of genetic and environmental factors and a specific combination is needed to initiate thyroid autoimmunity (2, 52, 53). Recently, substantial evidence on genetic factors influences on the association between AITDs and NTADs have been reported (54). Particularly, the evaluation of genetic risks factors for AITDs has demonstrated that some genes are exclusively implicated in GD or HT, conversely, others are involved in the pathogenesis of both GD and HT or AITDs and other ADs (55). Increasing evidence suggests that epigenetics mechanisms, including changes in DNA methylation, covalent modifications of histone tails, gene silencing mediated by non-coding RNA molecules, may be the link between genetics and environment, therefore epigenetic modifications of autoimmune-related genes, caused by environmental factors, would be implicated in the pathogenesis of ADs (56).

Thus, environmental factors, including high iodine intake, selenium deficiency and pollutants such as tobacco smoke, as well as infectious diseases and certain drugs, have been implicated in the development of AITD in genetically predisposed individuals (2, 52, 53). Among environmental factors, exposure to endocrine-disrupting chemicals (EDCs), including several natural compounds or synthetic chemicals, have been involved in ADs pathogenesis (56, 57). Prenatal, early and later-life exposures to EDCs insults may alter gene expression determining epigenetic modifications which play a significant role in the pathogenesis of ADs. In particular, several studies in cells and tissues of patients with AITDs, documented epigenetic modifications and consequent deregulation of gene expression levels, however their potential clinical implications and prognostic utility are still not completely clarified (56).

Moreover, the thyroid gland itself appears to play an important part in disease progression by interacting with the immune system, but the initial insult to thyroid cells, which activates the onset of AITD, remains unknown and seems to be strongly individual (2, 52, 53).

Although HT and GD have different phenotypes and the pathogenetic mechanisms leading to their dichotomy are unclear, both these conditions are generally believed to share a number of common etiological factors (58). In fact, there have been reports on monozygotic twins in whom one twin had HT and the other one had GD (59). Furthermore, both disorders may cluster in the same families or may coexist in the same glands and some patients may progress from one form to the other. According to many reports, in fact, the relative frequency of such a conversion from HT to GD or viceversa does not seem to be exceptionally low in both adults and children (10, 58, 60, 61) which suggests the existence of a continuum between GD and HT within the broad spectrum of AITDs. It is worthy to be underlined that the sequential shifting from HT to GD has been reported to occur more often in children and adolescents with DS than in those without DS (8, 9, 16, 37). These findings as a whole support the view that the association with DS might, *per se*, condition an over-expression of autoimmune phenomena (9), although the underlying mechanisms of these effects have not been clarified (62). However, the hypothesis of an extreme autoimmunity in DS patients is further supported by the non-exceptional co-occurrence of many ADs in the same DS individuals (62–65).

## Conclusions

In summary, it is likely to infer that the clustering of extra-thyroidal ADs in patients with HT and GD may be significantly conditioned by both patients' age at disease presentation and a possible association with DS. Another inference is that phenotypic expression of additional autoimmunity is not the same in patients with either GD or HT (44). Increasing evidence suggest that the interaction among genetics, environment and epigenetic modifications should determine a deregulation of gene expression and immune system response that, finally, seems to promote the pathogenesis of ADs through mechanisms still not completely clarified.

## Author Contributions

FD and MW conceived the review. GP, GS, and GZ were involved in literature search and prepared the figure. TA and DC drafted and wrote the manuscript. All authors approved the submitted version of the manuscript.

### Conflict of Interest Statement

The authors declare that the research was conducted in the absence of any commercial or financial relationships that could be construed as a potential conflict of interest.
